# Synergistic and Antagonistic Mechanisms of *Arctium lappa* L. Polyphenols on Human Neutrophil Elastase Inhibition: Insights from Molecular Docking and Enzymatic Kinetics

**DOI:** 10.3390/molecules30132764

**Published:** 2025-06-27

**Authors:** Yixun Sun, Mingbo Zhang, Yating Zhang, Yu Zheng, Jing Li, Qian Cai, Anqi Wang, Yang Qu

**Affiliations:** College of Pharmacy, Liaoning University of Traditional Chinese Medicine, Dalian 116600, China; 18940118579@163.com (Y.S.); mbzhang@126.com (M.Z.); rosezyt@163.com (Y.Z.); zhengyu1982@aliyun.com (Y.Z.); 15114120265@163.com (J.L.); 15265615851@163.com (A.W.)

**Keywords:** *Arctium lappa* L. polyphenols, human neutrophil elastase, synergistic mechanisms, molecular docking

## Abstract

This study systematically investigated the inhibitory mechanism of *Arctium lappa* L. polyphenols (ALP) against human neutrophil elastase (HNE). Molecular docking techniques were employed to predict the binding patterns and inhibition types between polyphenolic components and HNE, complemented by in vitro enzymatic tests to validate inhibitory efficacy. Combination index (CI) analysis was applied to evaluate synergistic effects. Through preliminary in vitro screening, chlorogenic acid, quercetin, and isochlorogenic acid A were identified as key bioactive constituents. Experimental results demonstrated that the half-inhibitory concentration (IC_50_) of individual compounds against HNE ranged from 46.4 to 203.3 μM, while ALP extract exhibited dose-dependent inhibition (IC_50_ = 0.99 mg/mL). Drug combination ratios based on individual IC_50_ values revealed synergistic effects (CI < 1) in chlorogenic acid-quercetin and isochlorogenic acid A-quercetin combinations, whereas antagonism (CI > 1) was observed in chlorogenic acid-isochlorogenic acid A pairs. The molecular docking results predicted that chlorogenic acid and isochlorogenic acid A competitively occupy the same binding site of the target protein (HNE) to exert inhibitory effects, thereby explaining the antagonism produced by their combination. In contrast, quercetin may inhibit HNE with a binding site different from that of chlorogenic acid or isochlorogenic acid A, which accounts for the observed synergistic effects. This study provides the first systematic elucidation of synergistic mechanisms of ALP as natural HNE inhibitors, providing theoretical foundations for developing novel natural HNE inhibitors with potential applications in acute lung injury, COVID-19-associated inflammatory conditions, and chronic inflammatory diseases.

## 1. Introduction

Human neutrophil elastase (HNE) is a major inflammatory protease primarily secreted by neutrophils in human blood leukocytes [1,2], belonging to the serine protease family. This enzyme plays a pivotal role in the innate immune system by combating pathogens and mediating inflammatory responses [3,4]. Under the physiological conditions, HNE exerts proteolytic activity through a catalytic triad composed of Ser195-Asp102-His57, thereby contributing to pathogen clearance and tissue remodeling. However, its dysregulated activity leads to extracellular matrix degradation and exacerbates inflammatory responses, which are closely associated with diseases such as acute respiratory distress syndrome (ARDS), pulmonary fibrosis, chronic obstructive pulmonary disease (COPD), and severe COVID-19 progression [4]. Although clinically used synthetic HNE inhibitors (e.g., sivelestat (SLT)) demonstrate efficacy in the treatment of such diseases [5], they always exhibit non-negligible adverse effects, including allergic reactions, hepatobiliary disorders, hematologic/lymphatic system abnormalities, and renal/urinary complications [6]. Therefore, discovering safer novel inhibitors from natural products is urgently needed.

Polyphenols are well-recognized bioactive compounds with anti-inflammatory and antibacterial activities [7,8]. In recent years, plant-derived polyphenols have emerged as a research focus due to their wide enzyme inhibitory activities [9,10,11,12]. For instance, many phenolics have been reported to have the potential to inhibit HNE [13]. The root of *Arctium lappa* L. (Asteraceae), commonly referred to as burdock root, is a dual-purpose herb widely utilized in both traditional medicine and culinary practices, exhibiting notable pharmacological properties including antibacterial, anti-inflammatory, and improving hyperlipidemia activities [14,15,16]. These pharmacological functions are attributed to its abundant polyphenolic constituents, including chlorogenic acid and quercetin, which possess notable anti-inflammatory and antibacterial activities [17]. Our preliminary study demonstrated that burdock root polyphenols can synergistically inhibit the activity of pancreatic lipase [18]. Quercetin has been confirmed to exhibit significant inhibitory activity against HNE [19,20,21,22]. Beyond the established HNE inhibitory activity of quercetin, further investigation should address two critical questions: (1) whether other polyphenolic constituents in the extract demonstrate comparable HNE suppression capabilities, and (2) if such compounds exhibit synergistic interactions in modulating HNE activity, a possibility that could significantly enhance therapeutic potential.

Molecular docking serves as a crucial tool in structural molecular biology and computer-aided drug design [23]. It has been extensively applied to study molecular interactions among protein, DNA, RNA, and natural or synthetic organic molecules [24]. The docking results can be utilized for virtual screening of pharmaceutical compounds [25]. In recent years, an increasing number of docking methodologies have become available, including Auto Dock Vina [26], Surflex [27], MOE [28], and Glide [29], providing diverse options for researchers.

In this study, the inhibitory effects of *Arctium lappa* root polyphenols (ALP) on HNE were evaluated through in vitro enzymatic activity assays. A combination index (CI) was employed to assess the synergistic inhibitory effects of polyphenol monomers in ALP. Molecular docking was performed to predict the inhibition modality using Auto Dock Vina, with subsequent analysis of key binding sites with Pymol.

## 2. Results and Discussion

### 2.1. Inhibitory Effect on HNE

#### 2.1.1. Inhibitory Effect of SLT on HNE Activity

**Figure 1:**
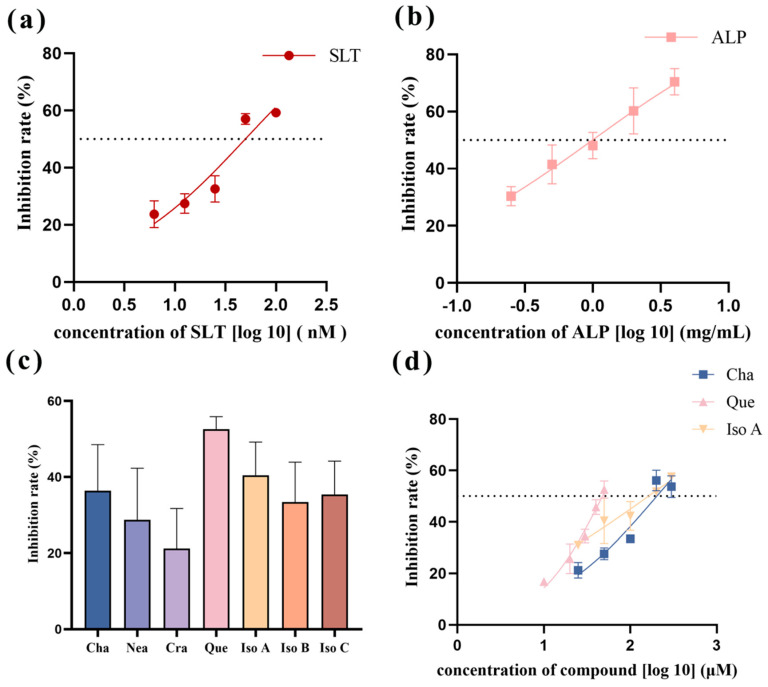
Nonlinear fitting curves for the actions of diverse compounds on HNE. (**a**) Nonlinear fitting curve for the action of sivelestat on HNE (SLT: sivelestat). (**b**) Nonlinear fitting curve for the action of *Arctium lappa* L. polyphenols on HNE (ALP: *Arctium lappa* L. polyphenols). (**c**) Bar graph of the inhibition rate of ALP single ingredients at a concentration of 50 μM. Cha: chlorogenic acid; Nea: neochlorogenic acid; Cra: cryptochlorogenic acid; Que: quercetin; Iso A: isochlorogenic acid A; Iso B: isochlorogenic acid B; Iso C: isochlorogenic acid C. (**d**) Nonlinear fitting curves of the effects of chlorogenic acid, quercetin, and isochlorogenic acid A on HNE (Cha: chlorogenic acid; Que: quercetin; Iso A: isochlorogenic acid A).

The concentration-dependent inhibitory effect of SLT on HNE exhibited progressive enhancement across the tested concentrations. Nonlinear regression analysis of the dose-response data (Figure 1a) yielded an inhibition curve with a calculated IC_50_ value of 49.76 nM (95% confidence interval: 39.52–66.32 nM). Notably, this value encompasses the previously reported IC_50_ of 44 nM for SLT-mediated HNE inhibition in reference [4], as evidenced by the overlapping confidence intervals, indicating no statistically significant difference between the two measurements.

#### 2.1.2. Inhibitory Effect of ALP on HNE Activity

As the concentration of ALP increases, its inhibitory effect on HNE was gradually enhanced. Through nonlinear fitting analysis (Figure 1b), the inhibition curve was drawn, and the IC_50_ value was calculated as 0.99 mg/mL.

#### 2.1.3. The Inhibitory Effect of Single Ingredients in ALP on HNE

The inhibitory effects of seven distinct single ingredients present in ALP, namely chlorogenic acid, neochlorogenic acid, cryptochlorogenic acid, quercetin, isochlorogenic acid A, isochlorogenic acid B, and isochlorogenic acid C, were individually examined for their capacity to inhibit HNE activity. The inhibitory rate of each compound was evaluated at an equimolar concentration of 50 μM in the standardized reaction system.

The corresponding results are shown in Figure 1c. It can be seen that quercetin is of the highest inhibition rate (52.6 ± 3.31%) on HME among the seven ingredients, followed by isochlorogenic acid A and chlorogenic acid with inhibition rates of 40.4 ± 8.78% and 37.0 ± 12.65%, respectively. The dose-dependent inhibitory effects of the three compounds were systematically evaluated using a five-point concentration series, with IC_50_ values calculated through nonlinear regression (Figure 1d). The IC_50_ values were determined as 203.3 μM for chlorogenic acid, 171.3 μM for isochlorogenic acid A, and 46.42 μM for quercetin. As far as we know, the inhibitory activities of chlorogenic acid and isochlorogenic acid are first reported herein.

#### 2.1.4. Combination Effects of Single Ingredients in ALP on HNE

**Figure 2:**
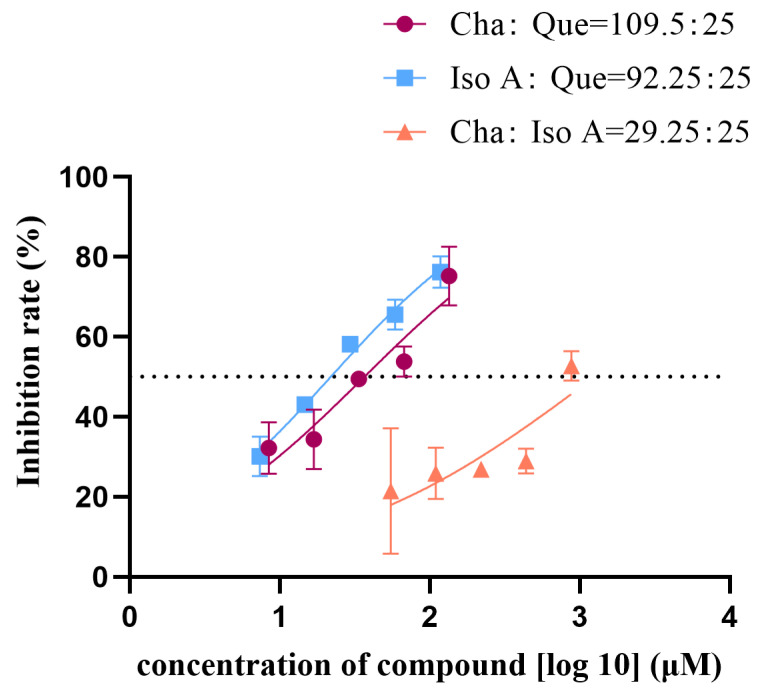
Nonlinear fitting curve of the combined effects of ALP single ingredients on HNE (Cha: chlorogenic acid; Iso A: isochlorogenic acid A; Que: quercetin).

**Figure 3:**
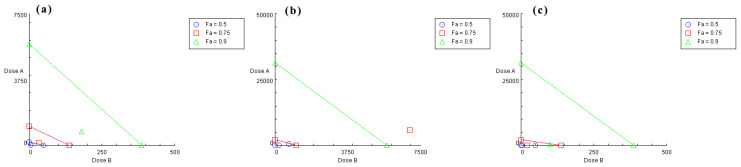
The isobolograms of combined effects on HNE. (**a**) Chlorogenic acid: quercetin (dose A: chlorogenic acid; dose B: quercetin). (**b**) Chlorogenic acid: isochlorogenic acid A (dose A: chlorogenic acid; dose B: isochlorogenic acid A). (**c**) Isochlorogenic acid A: quercetin (dose A: isochlorogenic acid A; dose B: quercetin). Combination data points on the diagonal line indicate an additive effect, while those on the lower left indicate synergism, and those on the upper right indicate antagonism (Fa: fraction affected).

Based on the IC_50_ values of chlorogenic acid, quercetin, and isochlorogenic acid A, the concentration ratios for the combinations effect assay were set as follows: chlorogenic acid:isochlorogenic acid A = 29.25:25; chlorogenic acid:quercetin = 109.50:25; isochlorogenic acid A:quercetin = 92.25:25. Each group contained five concentration gradients. The results were plotted as nonlinear fitting curves (Figure 2), where the x-axis represents the total concentration of drug combinations. The calculated IC_50_ values were 1253, 36.58, and 21.82 μM, respectively. The average inhibitory effect of each combination was recorded with CompuSyn 1.2 software [30] for analysis to calculate the combination index (CI). CI > 1 indicates antagonism, CI = 1 indicates additive effects, and CI < 1 indicates synergism. As shown in Table 1, both the chlorogenic acid-quercetin group and the isochlorogenic acid A-quercetin group exhibited CI values < 1, suggesting synergistic effects across all five concentrations compared to those of the single constituents. In contrast, the chlorogenic acid-isochlorogenic acid A group showed CI values > 1, indicating antagonistic effects in all five concentrations. The isobolograms (Figure 3) showed the relationship between Fa (fraction affected, representing enzyme inhibition rate) and CI values. Specifically, Fa = 0.75 corresponds to 75% enzyme inhibition. For the chlorogenic acid-isochlorogenic acid A combination (Figure 3b), the data points exhibited a distinct distribution pattern in the upper-right quadrant relative to the additive line, suggesting antagonistic rather than synergistic or additive effects on HNE inhibition. This observed antagonism might stem from steric hindrance caused by their substantial molecular volumes and competitive binding behavior at overlapping or proximal sites on the target protein. In contrast, both the chlorogenic acid-quercetin (Figure 3a) and isochlorogenic acid A-quercetin (Figure 3c) combinations showed points clustered in the lower-left quadrant of the additive line, demonstrating a synergistic effect. This may result from quercetin’s relatively smaller molecular size, which allows it to interact with more than one binding site with limited spatial constraints when combined with either chlorogenic acid or isochlorogenic acid A, thereby achieving complementary advantages.

### 2.2. Molecular Docking Between ALP Ingredients and HNE

#### 2.2.1. Docking Validation

**Figure 4:**
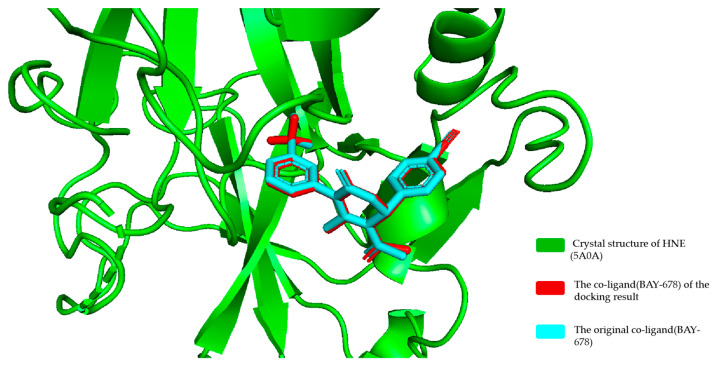
The alignment of the docked binding mode (red color) with the crystal structure (cyan color) of BAY-678.

To validate the reliability of the docking model employed, we redocked the co-ligand (BAY-678) from the crystal structure (PDB ID: 5A0A) into the target HNE. The resulting binding mode from docking was compared with that in the original crystal structure, as illustrated in Figure 4. The docked conformation aligned well with the crystal structure conformation, exhibiting a root mean square deviation (RMSD) of 0.93 Å. Furthermore, BAY-678 exhibits an inhibition constant Ki = 15 nM against HNE [31]. According to the thermodynamic formula, ΔG= −RTlnKi (where R = 1.987 × 10^−3^ kcal × mol^−1^ × K^−1^ and T = 298 K), the theoretical binding free energy is calculated as −10.67 kcal/mol. Our molecular docking prediction yielded a binding free energy of −9.511 kcal/mol, demonstrating excellent agreement with the theoretical value. The close agreement between the docked and crystallographic poses (RMSD = 0.93 Å) and the minor deviation in binding free energy (predicted: −9.511 kcal/mol vs. theoretical: −10.67 kcal/mol) confirm the accuracy of the docking protocol. This supports the model’s applicability for subsequent docking studies.

#### 2.2.2. Binding Modes of ALP Ingredients to HNE on the Active Site

To mechanistically interpret the combinatorial regulation of HNE activity by ALP constituents, molecular docking was performed to predict the binding sites and affinities to HNE. Molecular docking was carried out using Autodock Vina with a grid box centered on the ligand (BAY-678) of the crystal structure (5A0A.pdb). The docking scores of compounds to HNE, which is defined as the binding free energies by Autodock Vina, are listed in Table 2, together with the molecular structures. It can be seen that the binding free energies between the seven ingredients and HNE are all below −6.0 kcal/mol, indicating that the bindings of these ingredients to HNE are relatively stable. The binding free energy of quercetin to HNE is −6.538 kcal/mol, higher than that of the three isomers of isochlorogenic acid, which are in the range of −7.183 to −7.607 kcal/mol. Higher binding free energy indicates lower inhibitory activity in this case. This seems inconsistent with the above experimental results, which showed that quercetin is the strongest inhibitor among the ingredients considered. Previous studies [32] have demonstrated that, compared with large molecules, small molecules cannot fully occupy the active pocket due to their small sizes, which explains why quercetin exhibits inferior docking scores compared to the three isomers of sochlorogenic acid compounds but demonstrates better HNE inhibitory efficacy in in vitro enzymatic activity assays.

**Figure 5:**
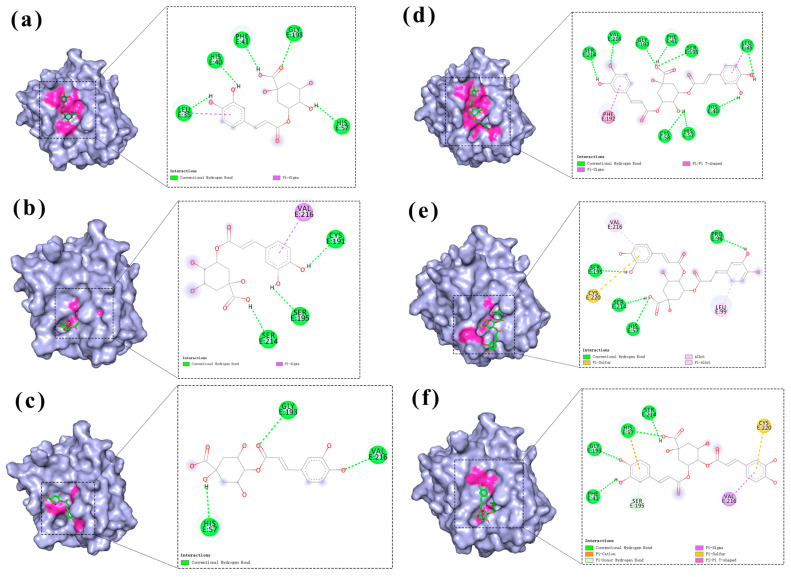
Binding mode of the effects of chlorogenic acid and its isochlorogenic acid isomers on the active site of HNE. (**a**) Chlorogenic acid. (**b**) Neochlorogenic acid. (**c**) Cryptochlorogenic acid. (**d**) Isochlorogenic acid A. (**e**) Isochlorogenic acid B. (**f**) Isochlorogenic acid C.

Binding site analysis was conducted using Discovery Studio Visualizer and Pymol for three caffeoylquinic acid isomers (chlorogenic acid, neochlorogenic acid, and cryptochlorogenic acid; Figure 5a–c) and three isochlorogenic acid isomers (isochlorogenic acid A, B, and C; Figure 5d–f). As shown in the figures, chlorogenic acid formed hydrogen bonds with Leu35, His40, Phe41, His57, and Gly193, demonstrating the strongest binding affinity. Neochlorogenic acid established hydrogen bonds with Cys191, Ser195, and Ser214, resulting in moderate binding strength. Cryptochlorogenic acid formed hydrogen bonds with His57, Gly193, and Val216, showing the weakest binding affinity among the three isomers. These findings were consistent with the molecular docking scores. Isochlorogenic acid A displayed the strongest binding affinity through hydrogen bonds with Leu35, His40, Phe41, His57, Cys58, Gly193, Ser195, Ser214, and Val216. Isochlorogenic acid C formed hydrogen bonds with Phe41, His57, Gly193, and Ser214, resulting in intermediate binding strength. Isochlorogenic acid B established hydrogen bonds with His57, Pro96, Ser195, and Ser214, demonstrating the weakest binding affinity among the three isomers. These observations correlated well with the molecular docking scoring results.

#### 2.2.3. Binding Modes of Chlorogenic Acid, Quercetin, and Isochlorogenic Acid A with HNE

The molecular docking using Autodock Vina with two different grid boxes, with details listed in Table 3, was performed with chlorogenic acid, quercetin, and isochlorogenic acid A as ligands. By comparing the differences in binding sites of identical compounds under two docking conditions, their inhibition types against HNE were inferred.

**Figure 6:**
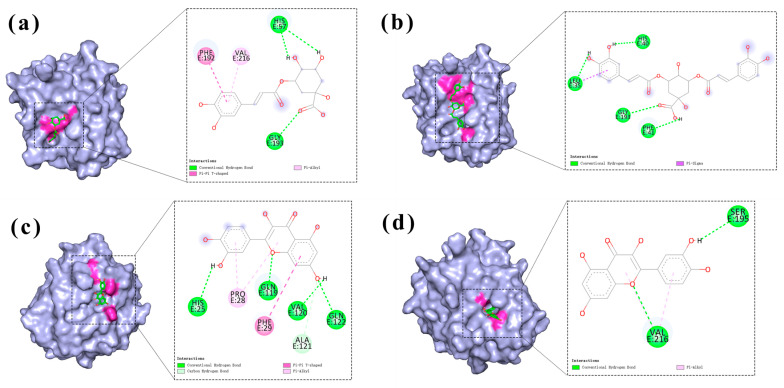
Diagram of the binding modes of chlorogenic acid, quercetin, and isochlorogenic acid A to the HNE at the binding sites of the whole protein, as well as the binding of quercetin to HNE at the active site. (**a**,**b**,**d**) Exploration of the binding sites of chlorogenic acid, quercetin, and isochlorogenic acid A to the entire protein of HNE. (**c**) The binding situation of quercetin at the original binding sites.

As shown in the comparative diagrams of HNE binding patterns under two different grid boxes (Figure 6), in the whole-protein binding site, chlorogenic acid (Figure 6a) formed hydrogen bonds with His57 and Gly193. Quercetin (Figure 6d) formed hydrogen bonds with His25, Gln119, Val120, and Gln122. Isochlorogenic acid A (Figure 6b) formed hydrogen bonds with Leu35, His40, Phe41, and Gly193. In the original binding site, quercetin (Figure 6c) formed hydrogen bonds with Ser195 and Val216. Notably, under both binding conformations, chlorogenic acid and isochlorogenic acid A consistently targeted the Gly193 site vicinity, suggesting their competitive occupation of identical or adjacent binding pockets on HNE. This spatial competition explains the antagonistic effects observed in combination therapy. In contrast, quercetin exhibited distinct binding localization without overlapping sites in different binding spaces. This unique binding topology enables quercetin to establish complementary binding mechanisms when co-administered with either chlorogenic acid or isochlorogenic acid A, ultimately leading to a synergistic therapeutic enhancement.

## 3. Materials and Methods

### 3.1. General Experimental Procedures 

ALP was obtained from the previous experiments of our laboratory [16]. HNE, SLT, N-Methoxysuccinyl-ALA-ALA-PRO-VAL P-Nitroanilide were purchased from Sigma-Aldrich Trading (Shanghai, China) Co., Ltd. Tris, DMSO were from Solarbio Science & Technology (Beijing, China) Co., Ltd. HCl, NaCl were from Kemiou Chemical Reagent (Tianjin, China) Co., Ltd. CH3COONa was from Xilong Chemical (Shantou, China) Co., Ltd. Chlorogenic acid (≥98%), neochlorogenic acid (≥98%), cryptochlorogenic acid (≥98%), quercetin (≥98%), isochlorogenic acid A (≥98%), isochlorogenic acid B (≥98%), and isochlorogenic acid C (≥98%) were from Sichuan Weikeqi Biotechnology (Chengdu, China) Co., Ltd. Purified water was from Wahaha Group (Hangzhou, China) Co., Ltd.

### 3.2. Experimental Methods

#### 3.2.1. Inhibitory Effect of SLT on HNE Activity

SLT was weighed and dissolved in DMSO, then diluted with buffer to prepare SLT solutions containing 5% DMSO with final concentrations of 100, 50, 25, 12.5, and 6.25 nM in the reaction system. These solutions were stored at 4 °C. The HNE inhibition experiment was performed with slight modifications according to the method described in reference [33]. Briefly, 30 μL of Tris-HCl buffer was added to 96-well plates and incubated at 37 °C with shaking for 5 min. Subsequently, 20 μL of HNE working solution with enzyme activity of 263 U/mL was added. The experimental groups were divided into a target drug group and a negative control group. The target drug group received 10 μL of drug solution, while the negative control group was treated with 10 μL of buffer containing 10% DMSO. After 5 min incubation at 37 °C with shaking, 40 μL of elastase substrate solution of N-Methoxysuccinyl-Ala-Ala-Pro-Val p-nitroanilide was added, and the absorbance at 405 nm was immediately measured, followed by subsequent measurements every 5 min. Each experiment was performed in triplicate, and the inhibition rate was calculated using Formula (1).

(1) $$Inhibition\;rate=\frac{K(negative\;control\;group)-K(target\;drug\;group)}{K(negative\;control\;group)}\times 100\%$$

The K (negative group) represents the reaction rate for the negative control group, and K (target drug group) denotes the reaction rate for the target drug group.

#### 3.2.2. The Inhibitory Effect of ALP on HNE Activity

ALP was weighed and dissolved in DMSO, then diluted with buffer to prepare ALP solutions containing 5% DMSO with final concentrations of 4, 2, 1, 0.5, and 0.25 mg/mL in the reaction system. These solutions were stored at 4 °C and analyzed using the same method described in Section 3.2.1.

#### 3.2.3. Inhibitory Effect of Single Ingredients in ALP on HNE

The ALP monomer compounds were weighed out and dissolved in 100% dimethyl sulfoxide (DMSO), then diluted with buffer solution to prepare five solutions of the ALP single ingredients containing 5% DMSO at different concentrations, respectively. These solutions were stored at 4 °C, and the analysis was conducted following the procedure described in Section 3.2.1.

#### 3.2.4. Combination Effect of Single Ingredients in ALP on HNE

Compounds with lower IC_50_ values in ALP were selected for further study. Their IC_50_ values were used as the proportion for combination therapy and analyzed using the same method described in Section 3.2.1. The combination effects were analyzed using the median-effect principle described by Chou-Talalay [34], and the combination index (CI) was calculated using CompuSyn software [30] to evaluate drug interaction effects. CI values were defined as follows: antagonism (CI > 1), additive effect (CI = 1), and synergism (CI < 1).

### 3.3. Molecular Docking for Predicting the Binding Mode of ALP Ingredients with HNE

The 3D structures of chlorogenic acid, neochlorogenic acid, cryptochlorogenic acid, quercetin, isochlorogenic acid A, isochlorogenic acid B, and isochlorogenic acid C were obtained from the PubChem database: https://pubchem.ncbi.nlm.nih.gov (accessed on 7 March 2025). The crystal structure of HNE (ID: 5A0A) was downloaded from the Protein Data Bank: http://www.rcsb.org (accessed on 11 June 2025). Structural pretreatment of the crystal structure of HNE was performed using Discovery Studio Visualizer, including deleting crystal water, chain B, and organic cocrystals. AutoDock Vina 1.2.3 was employed to define drug molecules as ligands and protein crystals as receptors, both saved in PDBQT format. Grid boxes were set according to the positions of native ligands and protein crystal dimensions (Table 3). Binding energies (kcal/mol) were defined as the lowest docking score produced with Vina, where values <−5.0 kcal/mol indicated strong binding and <−7.0 kcal/mol represented very strong binding [35].

### 3.4. Data Analysis

Each group of experiments was repeated three times. The results were expressed as the mean ± standard deviation (mean ± SD). The inhibition curves were plotted using ggprism, and the equivalent curves and combination index (CI) were obtained by CompuSyn software [30].

## 4. Conclusions

This study investigated in vitro the inhibitory effects of ALP and its main single ingredients on HNE. The results demonstrated that ALP exhibited inhibitory activity against HNE with an IC_50_ of 0.99 mg/mL. At equivalent concentrations, the main ALP single ingredients, including chlorogenic acid, quercetin, and isochlorogenic acid A, showed higher HNE inhibition rates with IC_50_ values of 203.3 μM, 46.42 μM, and 171.3 μM, respectively. Combination of chlorogenic acid with quercetin, and isochlorogenic acid A with quercetin showed synergistic inhibition on HNE, whereas the combination of chlorogenic acid and isochlorogenic acid A exhibited antagonistic effects. Molecular docking analysis revealed that chlorogenic acid and isochlorogenic acid A competitively bind to identical or adjacent catalytic sites on HNE, while quercetin exhibits a distinct binding pattern within alternative binding cavities without spatial overlap. This finding elucidates the differential pharmacological outcomes of three combination regimens: the antagonistic interaction between chlorogenic acid and isochlorogenic acid A arise from their competitive occupation of adjacent binding pockets, whereas the synergistic effects observed in quercetin-containing combinations stem from its binding sites distinct from other compounds that enables its mutually beneficial interactions with either chlorogenic acid or isochlorogenic acid A by binding simultaneously with HNE.

In addition to burdock (*Arctium lappa* L.), many herbs which are rich in polyphenols, possess anti-inflammatory effects. Our research revealed that the polyphenolic compound in ALP exhibits inhibitory activity against HNE synergistically. There are several herbs, such as wild honeysuckle flower and Caulis Lonicerae Japonicae, which are also rich in polyphenolics, that may be the potential inhibitors of HME. This study expands the possibilities for obtaining novel HNE inhibitors from natural medicinal sources. However, this study only demonstrated the synergistic HNE inhibition of ALP in vitro. Further research is needed to validate these effects through in vitro and in vivo experiments to elucidate the underlying mechanisms.

## Figures and Tables

**Table 1:** The inhibitory effects of chlorogenic acid and quercetin, isochlorogenic acid A and quercetin, chlorogenic acid, and isochlorogenic acid A, and their combination index (CI).

Combination	Groups	Dose A (μM)	Dose B (μM)	Inhibition Rate (%)	CI
chlorogenic acid (A): quercetin (B)	1	6.83	1.56	32.23 ± 6.45	0.18 ± 0.07
	2	13.66	3.13	34.40 ± 7.45	0.32 ± 0.16
	3	27.31	6.25	49.47 ± 1.85	0.27 ± 0.02
	4	54.63	12.50	53.77 ± 3.75	0.44 ± 0.08
	5	109.25	25.00	75.23 ± 7.39	0.29 ± 0.13
isochlorogenic acid A (A): quercetin (B)	1	5.77	1.56	21.40 ± 4.94	0.37 ± 0.17
	2	11.53	3.13	43.00 ± 1.91	0.22 ± 0.03
	3	23.06	6.25	58.10	0.16
	4	46.13	12.50	65.53 ± 3.75	0.20 ± 0.05
	5	92.25	25.00	76.23 ± 3.98	0.21 ± 0.06
chlorogenic acid (A): isochlorogenic acid A (B)	1	29.75	25.00	21.47 ± 15.66	24.3 ± 35.54
	2	59.50	50.00	25.83 ± 6.45	6.60 ± 4.70
	3	119.00	100.00	26.87 ± 1.85	9.55 ± 1.78
	4	238.00	200.00	28.93 ± 3.10	15.74 ± 5.08
	5	476.00	400.00	52.67 ± 3.70	3.98 ± 1.25

**Table 2:** The docking scores of HNE with the ingredients of ALP.

Number	Name	Structure	Score (kcal/mol)
1	Chlorogenic acid	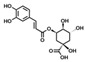	−7.278
2	Neochlorogenic acid	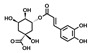	−7.092
3	Cryptochlorogenic acid	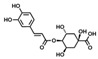	−6.284
4	Quercetin	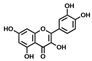	−6.583
5	Isochlorogenic acid A	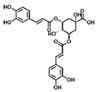	−7.607
6	Isochlorogenic acid B	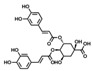	−7.183
7	Isochlorogenic acid C	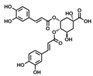	−7.511

**Table 3:** The detailed parameters for the two docking approaches.

Docking Conditions	Center X	Center Y	Center Z	Size X	Size Y	Size Z	Spacing
active site	−6.518	31.860	−3.711	40	40	40	0.375
whole-protein	−14.42	28.644	1.197	90	96	92	0.503

## Data Availability

All the data are shown in the manuscript.

## Abbreviations

The following abbreviations are used in this manuscript:

ALP	*Arctium lappa* L. polyphenols
HNE	Human neutrophil elastase
CI	Combination index
IC_50_	The half-inhibitory concentration
ARDS	Acute respiratory distress syndrome
COPD	Chronic obstructive pulmonary disease
SLT	Sivelestat
Cha	Chlorogenic acid
Nea	Neochlorogenic acid
Cra	Cryptochlorogenic acid
Que	Quercetin
Iso A	Isochlorogenic acid A
Iso B	Isochlorogenic acid B
Iso C	Isochlorogenic acid C
Fa	Fraction affected
